# Not mismatch, but absence: the structural inequity of seasonal influenza care in low-income and sub-Saharan African countries

**DOI:** 10.31744/einstein_journal/2026CE2549

**Published:** 2026-09-18

**Authors:** Mauer Alexandre da Ascensão Gonçalves, Humberto Morais, Howard Lopes Ribeiro

**Affiliations:** 1 Center for Advanced Studies in Medical Education and Training Faculty of Medicine Agostinho Neto University Luanda Angola Center for Advanced Studies in Medical Education and Training, Faculty of Medicine, Agostinho Neto University, Luanda, Angola.; 2 Cardiology Department Hospital Militar Principal Instituto Superior Luanda Angola Cardiology Department, Hospital Militar Principal Instituto Superior, Luanda, Angola.; 3 Center for Research and Drug Development Universidade Federal do Ceará Fortaleza CE Brazil Center for Research and Drug Development, Universidade Federal do Ceará, Fortaleza, CE, Brazil.; 4 Post-Graduate Program in Pathology Universidade Federal do Ceará Fortaleza CE Brazil Post-Graduate Program in Pathology, Universidade Federal do Ceará, Fortaleza, CE, Brazil.; 5 Post-Graduate Program in Translational Medicine Universidade Federal do Ceará Fortaleza CE Brazil Post-Graduate Program in Translational Medicine, Universidade Federal do Ceará, Fortaleza, CE, Brazil.

Dear Editor,

Seasonal influenza should not be understood as a series of isolated outbreaks, but rather as a predictable, cyclical public health challenge that recurs annually and has disproportionate consequences in regions with limited healthcare capacity. Despite this recurring burden, seasonal influenza remains a critically overlooked driver of morbidity and mortality in sub-Saharan Africa. Global estimates suggest that up to 650,000 annually result from influenza-associated respiratory illness, with a disproportionate burden falling on resource-constrained settings.^(1)^ In Africa, children under five account for a substantial proportion of severe outcomes, with an estimated 34,000–65,000 deaths annually. Pregnant women, older adults, and people living with HIV also face heightened risks.^(1,2)^ The regional under-5 mortality rate of 94.8 per 100,000 person-years remains markedly above the Global Action Plan target of 60 deaths per 100,000 person-years.^(1)^

Despite its predictable recurrence, influenza continues to be structurally deprioritized within national immunization programs and pandemic preparedness strategies across the region.^(1)^ Vaccination coverage among the general population in sub-Saharan Africa remains negligible, and fewer than 10% of countries maintain functional seasonal influenza policies. Persistent structural barriers, including fragile cold-chain systems and limited domestic investment, have normalized reliance on natural infection as a surrogate for immunization. As a result, health systems remain chronically exposed to recurrent influenza peaks compounded by ongoing viral evolution.^(2)^

The emergence of influenza A(H3N2) subclade K in 2025, reported in the United States, highlights the impact of antigenic drift and the limitations of control strategies that rely predominantly on vaccine strain matching.^(2,3)^ While such mismatches can compromise vaccine effectiveness even in well-resourced settings, their effects in low-resource settings are further exacerbated by structural gaps in vaccine access and delivery.^(2,3)^ Identified months after vaccine strain selection, this variant carries mutations in the hemagglutinin gene that may compromise vaccine effectiveness.^(3)^ Yet, in sub-Saharan Africa, the central issue is not mismatch but absence. Even in seasons with an optimal vaccine match, delayed procurement, limited distribution, and minimal uptake negate the potential benefits at the population level.

Furthermore, surveillance gaps continue to undermine timely responses. Although the Global Influenza Surveillance and Response System (GISRS) has expanded geographically, its capacity to translate virological data into timely public health action remains uneven.^(2)^ Consequently, influenza activity is frequently detected only after peak transmission, when healthcare systems are already overwhelmed. These dynamics underscore a critical misalignment in global influenza preparedness: while high-income countries - mobilize resources in response to seasonal anomalies, low-income regions face a continuous, unmitigated burden. Influenza, therefore, should be reframed not as a seasonal inconvenience, but as a persistent and structurally neglected health threat.

Addressing this persistent burden requires sustained investment in system-level resilience rather than episodic responses. Strengthening syndromic surveillance within primary health- care, improving clinical management pathways, and expanding equitable access to preventive and therapeutic interventions are essential.^(2)^ In contexts where vaccines remain unavailable, non-pharmaceutical strategies, such as improved ventilation and community-based isolation, represent pragmatic and cost-effective measures.^(2,3)^ Additionally, integrating the early use of antiviral therapies into clinical protocols could substantially reduce morbidity and mortality, even in the absence of widespread diagnostic capacity.^(2)^

The lack of prioritization of influenza is also evident in national policy frameworks. Angola’s recent policy developments further illustrate this misalignment.^(4)^ The National Immunization Strategy (NIS) 2026–2030, approved in November 2025, sets ambitious targets, including achieving 90% national vaccination coverage, reducing zero-dose the proportion of children to below 5%, eliminating neonatal tetanus, while sustaining polio-free status and advancing measles elimination.^(4)^ Supported by the WHO and Gavi,^(5)^ the strategy prioritizes cold chain expansion, workforce training, sustainable financing, and the integration of immunization into primary health care. However, despite its comprehensive scope and forward-looking design, influenza remains notably absent from its core priorities, reinforcing the persistent gap between the global influenza burden and national immunization agendas in low-income settings.

Ultimately, the recurring visibility of influenza surges in high-income countries should not obscure the chronic, under-recognized burden experienced elsewhere. In sub-Saharan Africa, vulnerability is driven less by viral evolution than by persistent inequities in access, infrastructure, and policy prioritization (Figure 1).^(1)^ Recognizing influenza as a persistent and recurring public health challenge is essential to advancing equitable preparedness and reducing the global impact of respiratory infections.

**Figure 1:**
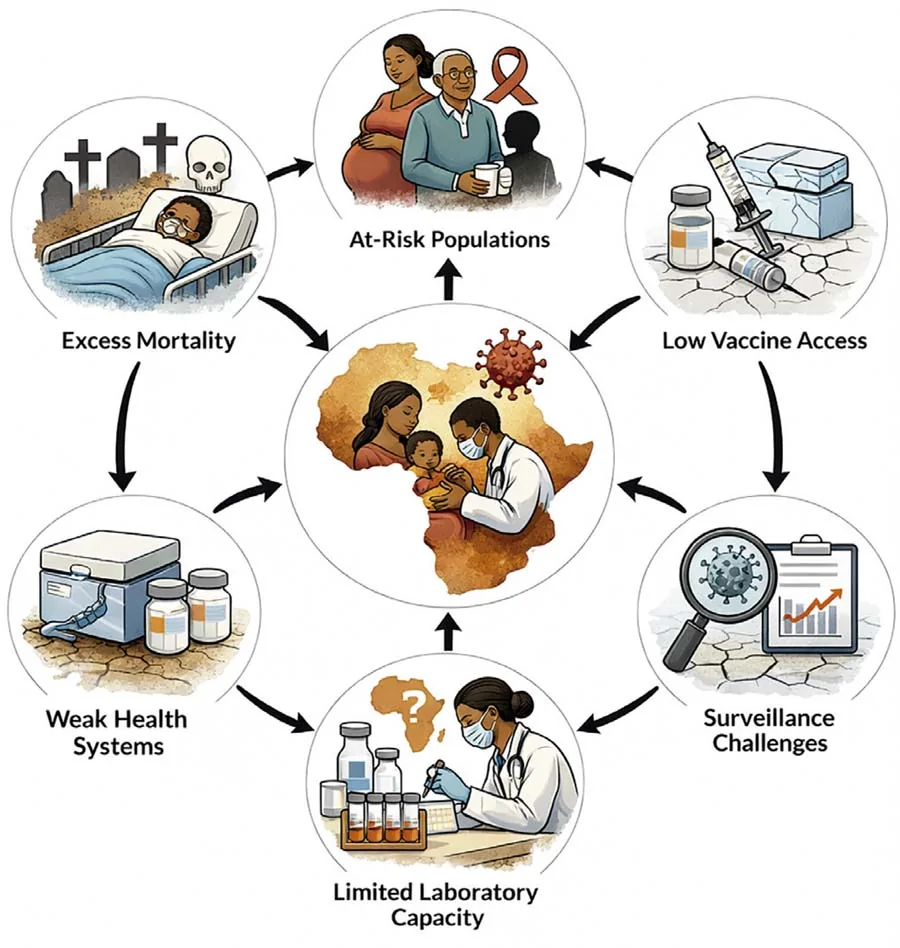
Schematic representation of the persistent and structurally neglected burden of seasonal influenza in sub-Saharan Africa. The central panel highlights the regional impact, while surrounding elements illustrate key interconnected drivers: high-risk populations (children under five, pregnant women, older adults, and people living with HIV), excess mortality, limited vaccine access, surveillance gaps, weak health systems, and constrained laboratory capacity. Together, these factors sustain a cycle of recurrent transmission and inadequate response, underscoring the need for strengthened surveillance, equitable vaccine access, and system-level resilience
